# Corrigendum

**DOI:** 10.1002/ccr3.6648

**Published:** 2022-11-27

In the article entitled “Large papillary thyroid cancer bed recurrence causing overlying skin ulceration and bleeding”^1^ that was previously published in Volume 10 Issue 7 of Clinical Case Reports, the last author's name was incorrect in the published version.

The correct author list should be as follows: Konstantinos A. Boulas, Maria Nathanailidou, Konstantinos Sitaridis, Isaac Filippidis, Ioannis Tsiariglis, Iliana Domi, Anestis Hatzigeorgiadis.

The publisher apologizes for the error.
